# Low energy cost for cultured pearl formation in grafted chimeric *Pinctada margaritifera*

**DOI:** 10.1038/s41598-018-25360-5

**Published:** 2018-05-14

**Authors:** Gilles Le Moullac, Claude Soyez, Chin-Long Ky

**Affiliations:** Ifremer, UMR 241 Ecosystèmes Insulaires Océaniens (EIO), Labex Corail, Centre du Pacifique, BP 49, 98719 Taravao, Tahiti French Polynesia

## Abstract

The pearl oyster is one of the rare animal models that support two distinct genomes, through the surgical graft process operated for culture pearl production. This grafted organism is assimilated to a chimera whose physiological functioning remains poorly known. The question of the energy expenditure comparison between chimera and non-chimera animals arises. To answer this question, grafted and non-grafted pearl oysters were evaluated for their energetic needs by the indirect calorimetry method. This method made it possible to measure the energy expenditure based on the respiration rate (RR) measurement, reflecting the basal metabolism. The results showed that the RR values for grafted and non-grafted pearl oysters were not significantly different (*p* < 0.05). The estimated cost of pearl calcification including CaCO_3_ and proteins synthesis was 0.237 ± 0.064 J h^−1^, representing 0.64% of the total energy expenditure of grafted pearl oysters. This study made it possible, for the first time, to see the energy cost of cultured pearl formation in *P. margaritifera* and the little impact in the energetic metabolism of the chimera organism.

## Introduction

The grafting process during cultured pearl formation creates a unique organism, a chimera from a genetic point of view, that consists of genetic material delivered by two distinct genomes: the graft of the donor into the gonad called pearl pouch of a recipient oyster^1–3^. This grafting operation is not the substitution of one organ by another, but will lead to the creation (formation) of a new organ, the pearl sac whose genome is derived from the graft donor, this one will implant by forming vascular connections in a symbiotic relationship. The internal structure of the pearl pouch is largely conjunctive and muscular and is traversed by sinuses and haemolymphatic vessels. An extracellular matrix is formed from haemocytes that evolve into conjunctive cells. The spongy and connective environment keeps the pearl sac and promotes the pearl formation without constraints^4,5^. The pearl sac, through the epithelium, develops^4^, secretes and deposits the nacreous layers onto the nucleus to produce a pearl after 15 to 20 months of culture^5^. This new organ will therefore consume energy to develop and synthesize the proteins of biomineralization^6^. During the entire period of pearl formation, the pearl sac receives energy from the nutrients provided by the recipient oyster from the food it filters. Cultured pearl growth is linear. Its rate has recently been estimated in *P. margaritifera* in six grow-out locations in French Polynesia and ranges between 0.051 and 0.064 g per month^7^, which corresponds to an average daily nacre deposition thickness of approximately 3.4 µm per day^8^. This growth therefore requires a constant energy supply during cultured pearl formation. The role played by the recipient oyster in pearl growth is thus determinant, as highlighted by the positive correlation of pearl size with oyster shell height and total weight over the culture period^9^. In addition, pearl growth potential is also indirectly controlled by the graft biomineralization capabilities, as a significant donor effect has been found for pearl size determination in wild^10^ and hatchery-produced donors^11^, based on the expression levels of some mantle genes such as Pif-177^12^.

Earlier studies in calcifying marine invertebrates including mollusks estimate that biomineralization is an energetically costly process. In molluskan shells consisting of inorganic crystals (calcite and/or aragonite) and an organic (mostly proteinaceous) matrix, production of the organic matrix was proposed to be the main cost-intensive process in shell growth^13^. Compared to the estimated total cost for inorganic shell material (1–2 J mg^−1^ CaCO_3_)^14,15^, total costs for protein synthesis for shell formation are much higher (29 J mg^−1^)^16^. Finally, the protein content (soluble and insoluble) would be between 3 and 5% of the weight of shellfish shells^13^. All of this information gathered on the formation and composition of shellfish shells should be able to provide a conservative estimate of the cost of the pearl to estimate energy requirements during pearl formation in grafted pearl oysters.

Better knowledge on the interplay between recipient and donor oysters, in relation to pearl growth remains one of the most important challenges for the pearl industry in order to improve cultured pearl size. In particular, we need to ask whether the functional pearl sac increases the overall energy expenditure of the recipient pearl oyster taking into account their reproductive state. To answer this question, an experimental graft was designed followed by an estimation of the extra energy costs for pearl sac functioning in grafted recipient pearl oysters. The energy expenditure of grafted and non-grafted pearl oysters of the same age was measured to determine the energy cost differences. The energy expenditure was assessed using an indirect calorimetry method based on the measurement of the respiration rate of starved pearl oysters, reflecting the standard metabolic rate taking into account the condition index as indicator for maintenance and sexual activity of molluscs^17^.

## Results

### Comparison of standardized RR in grafted vs. non-grafted recipient pearl oysters

The standardized RR values were 0.595 ± 0.107 mg O_2_ h^−1^ g^−1^ (N = 20) for the grafted pearl oysters and 0.604 ± 0.080 mg O_2_ h^−1^ g^−1^ (N = 20) for the control pearl oysters. The CI mean value were 0.0255 (N = 20) for the grafted pearl oysters and 0.0259 (N = 20) for the control pearl oysters. ANCOVA indicated that there was no significant effect of the graft on RR (F = 0.055, p = 0.816) and of the covariate CI on RR (F = 0.110, p = 0.742). As well as the interaction RR x CI was not significant (F = 2.568, p = 0.117). Data are presented in Table S1.

Within the grafted pearl oysters batch, our results showed there was no effect of donor origin on RR (F = 0.456, p = 0.717). See the experimental grafting design in Table S2.

### Pearl harvest and weighing

After each series of oxygen consumption measurement, the pearls were collected and weighed. Only 17 pearls were harvested due to the presence of one keshi instead of a pearl that was excluded of the analysis. The final mean pearl weight was 1.50 ± 0.25 g, but the mean deposited nacre weight, found by deducting the nucleus weight, was 0.91 ± 0.25 g.

### Nacre deposited was correlated to shell recipient oyster but not to RR

The deposited weight of nacre was correlated with that of the recipient oyster shell (ddl = 17, r = 0.525), but not at the RR (ddl = 17, r = 0.221).

### Assessment of the proportion of energy allocated to the formation of the pearl

Taking into account the whole culture duration, between 441 and 476 days, the mean rate of nacre deposition on pearls was 0.08 ± 0.02 mg h^−1^. The nacre was composed of CaCO_3_ and proteins, and their respective contribution in nacre formation was of 95% and 5%, or 1.5 J mg^−1^ for CaCO_3_ and 29 J mg^−1^ for proteins. Therefore, the energy cost of CaCO_3_ and proteins synthesis was 0.117 ± 0.032 J h^−1^ and 0.119 ± 0.032 J h^−1^ respectively. Hence, the rate of energy cost for nacre deposition on the pearl was 0.237 ± 0.064 J h^−1^ while the mean energy expenditure of individuals calculated from the RR was 38.64 ± 8.45 J h^−1^ for the grafted pearl oysters. This means that a pearl would cost 0.64 ± 0.23% of the energy expenditure of the grafted pearl oysters.

## Discussion

As the main result, our study showed that the basal metabolic rate of the grafted pearl oysters was the same as that for the non-grafted pearl oysters. No supplementary energy cost is then needed in the grafted chimera organism. The part of energy allocated to the pearl sac was statistically not detectable by the indirect calorimetry method.

For cultured pearl formation, energy of the recipient pearl oysters is potentially allocated to the functioning of the pearl sac epithelial cells. These cells were first known to be implicated in the molecular expression of the matrix protein synthesis, which is involved in nacre production^6,18,19^. Second, the pearl sac may ensure pearl rotation, thereby determining overall pearl shape. During culture time, the pearl being formed is continuously in movement because it turns. When the rotation follows a random movement, the pearl is round. In contrast, if the rotation takes place on a single axis, the pearl will have a teardrop or ringed shape^20^. Regarding the main question about the amount of energy allocated to both the formation and rotation of the pearl, our result clearly showed that this was insignificant, based on the energy expenditure (respiration rate) measurement of grafted *vs*. non-grafted recipient pearl oysters. Consequently, the pearl being formed in the pearl sac did not have an impact on the energy metabolism of the recipient pearl oysters. The metabolic rates were regulated by temperature and food availability^21^. These two parameters affected pearl growth, which is significantly correlated with the weight and height (dorsal-ventral measurement) of the recipient shell^7^, as confirmed in this study. Part of the energy may be allocated to pearl rotation. Current knowledge does not suggest otherwise, i.e., that the rotation is a self-organized phenomenon caused and sustained by physical forces on the pearl surface on which the nacre grows: a kind of natural ratchet^22^. In other words, the rotation of the pearls was based on a physical and chemical phenomenon.

Although the instant energy cost was low at the individual scale, it was interesting to try to find this value for the whole cultured pearl formation duration. First, the results showed that the rate of nacre production during the culture time was 1.98 mg per day or 0.08 mg per hour. Second, the calcification cost in marine invertebrates is known to be 1–2 J mg^−1^ of CaCO_3_ in mollusks^14^, this is also estimated at 1.52 J mg^−1^ in coral^15^. Moreover the protein synthesis cost was estimated to be of 29 J mg^−1^ proteins^13^. Application of this range of CaCO_3_ and proteins cost to nacre biosynthesis revealed that the total nacre production cost during pearl formation would be 2.6 kJ for almost 15 months and a half of the culture time, while energy expenditure was 426.6 kJ during this period.

This energy cost did not seem to be affected by the donor or recipient pearl oysters. In fact, the results showed no family donor effect nor reproductive state of recipient pearl oysters on the respiration rate measurement. These results failed to reveal a differential metabolic level attributable to differences in pearl sac activities among donor pearl oysters of different origins. This seems to contradict the previous finding of a donor effect on cultured pearl size in wild oysters^10^, but highlights the complex interplay and regulation between the donor and recipient in terms of pearl size realization. Further studies should be designed to monitor pearl oyster growth at the family scale between lines selected for growth performance, and should definitely conclude on an eventually re-allocation of energy toward pearl sac at constant energy expenditure. This study makes it possible for the first time to suggest that the energy cost for culture pearl formation is low and even insignificant for the recipient pearl oyster in *P. margaritifera*. The energy expenditure comparison between chimera and non-chimera organism was then similar, reflecting the perfect integration of the foreign genome in the recipient oyster.

## Material and Methods

### Biological material and grafting design

*Pinctada margaritifera* pearl oysters (N = 120), obtained in the wild and used as recipient, were grafted at a commercial pearl farm operated by the Pahai Poe company on the atoll of Apataki (Tuamotu Archipelago, French Polynesia) in August 2015. The grafting operation was conducted by an expert^23^. The nuclei used for this purpose were made from the shells of freshwater mussels and measured 2.4 BU in size (6.054 mm in diameter, 591 mg in weight - Nucleus Bio, Hyakusyo Co. Japan).

Four origins of pearl oysters were used as donors (10 grafts per donor). Three were originated from the atolls of Ahe (A), Apataki (B), and Takapoto (C) (Tuamotu Archipelago), where they were caught as spat and were reared in Apataki atoll during the juvenile stage. A fourth (E) was used consisting of hatchery-produced spat to provide another 120 grafted pearl oysters (10 grafts per donor). Experimental grafting design is presented in Table S2. At checking time (45 days post-graft), 104 recipient oysters had successfully retained their nuclei (four oysters died and 12 had rejected their nuclei by 40 days post-graft). These 104 oysters were transferred by plane to Tahiti in December 2015, together with 40 ungrafted pearl oysters that originated from the same recipient pool.

### Respiration rate (RR) measurements

In August 2016, the energy expenditure was estimated on 40 pearl oysters (20 grafted and 20 ungrafted) over a 35 day trials, corresponding to a range of 441 to 476 days post-graft. The pearl oysters were transferred from the lagoon to the ecophysiological measurement system (EMS), where they were individually placed in a metabolic chamber to monitor their oxygen consumption without food supply for 48 hours. The EMS is composed of five open-flow chambers; four oysters were placed in four chambers and the remaining fifth chamber was left empty to serve as a control^21^. The drains of the chambers are each equipped with a two-way electromagnetic valve activated by an automaton. When the valve of one measuring chamber was opened, the released water was analyzed for 3 min using an oximeter (OXI 358, WTW, Weilheim, Germany) to measure the dissolved oxygen (the data were stored on a computer). Each cycle was completed within 3 min and another cycle started in the control chamber for 3 min (sequence: chamber 1, control, chamber 2, control, etc.). The specimens remained in the chambers for 48 h and measurements were taken every 24 min until 120 oxygen consumption measurements had been recorded. The RR, expressed in mg O_2_ h^−1^, was estimated from the difference in the oxygen concentration between the control and experimental chambers using the following formula: *RR* = *V (O*_1_ − *O*_2_), where *O*_1_ is the oxygen concentration in the control chamber, *O*_2_ is the oxygen concentration in the experimental chamber, and *V* is the water flow rate. Ten oxygen consumption measurement series were conducted in groups of four pearl oysters composed each time of two grafted and two non-grafted pearl oysters. The energy expenditure was expressed as RR of individuals (total energy expenditure) and as standardized RR (see 2.4 section).

### Pearl harvest and Condition index

**Figure 1:**
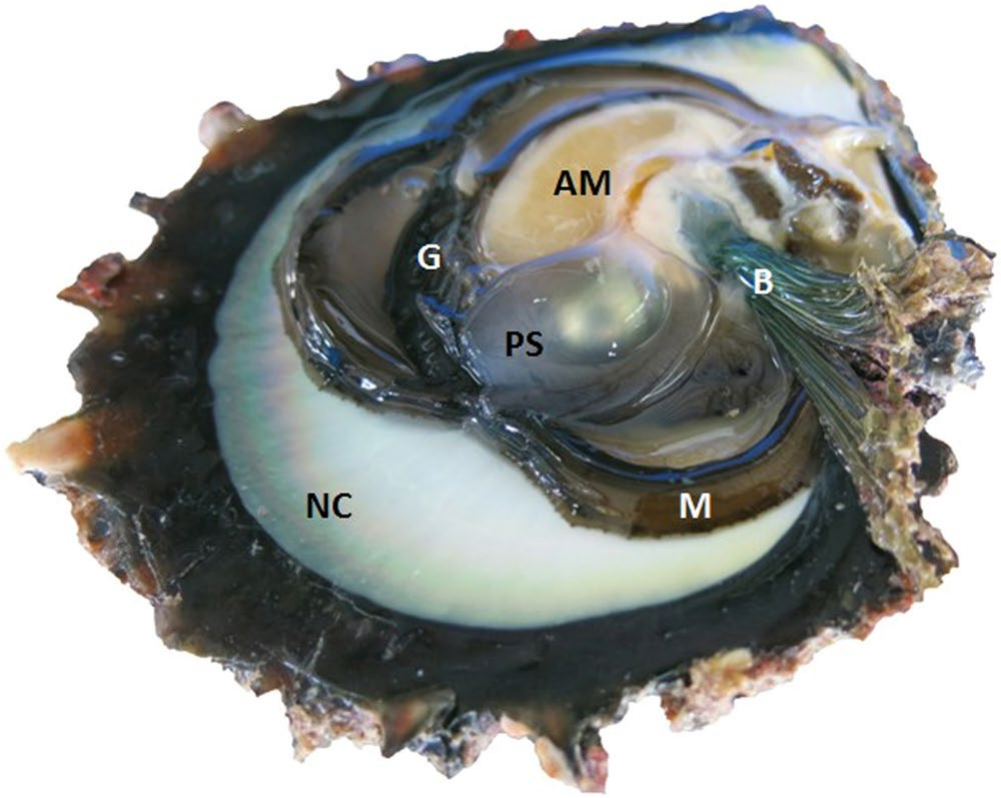
Recipient pearl oyster from *Pinctada margaritifera* with one shell valve removed showing the general anatomy and the cultured pearl inside the chimera organ, the pearl sac: AM, adductor muscle; NC, nacreous zone; G, gills; B, byssus; M, mantle; and PS, pearl sac.

At the end of RR measurement, pearl oysters were open by cutting the adductor muscle with a blade. In grafted pearl oysters, pearl was harvested by cutting the pearl sac (Fig. 1). The pearls were weighed using an analytical balance (AS60/220.R2, Radwag, Poland). Then, the shell and flesh were freeze dried, and then weighed using the same balance. The condition index was computed as the ratio of dry flesh weight to dry shell weight.

### Standardization

It was necessary to correct for differences in specimen weight. Pearl oysters were dissected in order to freeze dry (Christ Martin, Germany) the flesh to obtain all of the individual dry weights. Oxygen consumption values were converted to a standard animal basis (1 g, dry weight) using the formula $Ys={(\frac{Ws}{We})}^{b}\times Ye$, where *Ys* is the physiological activity of a standard oyster, *Ws* is the dry weight of a standard oyster (1 g), *We* is the dry weight of the specimen, *Ye* is the measured physiological activity (the total, and b is the allometric coefficient of 0.75 for the oxygen consumption rate^24^.

The energy expenditure was transformed into Joules (J), with the following conversion: 14.1 J for 1 mg O_2_^25^ and 1.5 J for 1 mg CaCO_3_ for mollusk^14^ and coral^15^.

### Statistics

The normality of the data distribution and homogeneity of the RR variance were tested with the Shapiro-Wilk test and Bartlett’s test, respectively. The RR data fulfilled conditions for parametric tests. As a proportion data, Condition Index (CI) was arcsine square root transformed. Comparisons were made using ANCOVA for the comparison of RR of grafted and non-grafted pearl oysters as CI as covariate which represent the recipient pearl oyster effect. One-way ANOVA was used to compare the RR means of grafted pearl oysters according to the graft donor family (four families).

## Electronic supplementary material

Dataset1, Dataset2
