# Supplementary material for: Low energy cost for cultured pearl formation in grafted chimeric Pinctada margaritifera
Source: Sci Rep. 2018 May 14;8:7520. doi: 10.1038/s41598-018-25360-5 (PMC5951858; doi:10.1038/s41598-018-25360-5)
Supplement: Supplementary file 1 — Dataset1, Dataset2 [file 41598_2018_25360_MOESM1_ESM.doc]

**Low energy cost for cultured pearl formation in grafted chimeric *Pinctada margaritifera***

Gilles Le Moullac*, Claude Soyez, Chin-Long Ky

Data set 1 : Physiological measurements in grafted and non grafted pearl oysters.

| Series | Recipient  oyster n° | Treatment | Shell weight (g) | Condition index (CI) | O_2_ consumption (mg h ^-1^ g ^-1^) | Pearl nacre weight (g) |
| --- | --- | --- | --- | --- | --- | --- |
| 1 | 1 | control | 173.2 | 0.026 | 0.656 |  |
|  | 2 | control | 226.6 | 0.021 | 0.426 |  |
|  | 3 | grafted | 193.9 | 0.025 | 0.528 | 0.9813 |
|  | 4 | grafted | 234 | 0.024 | 0.674 | 1.1592 |
| 2 | 5 | control | 162.9 | 0.026 | 0.736 |  |
|  | 6 | control | 199 | 0.025 | 0.569 |  |
|  | 7 | grafted | 185.8 | 0.027 | 0.408 | 1.0464 |
|  | 8 | grafted | 177.8 | 0.029 | 0.644 | 0.6641 |
| 3 | 9 | control | 148.8 | 0.031 | 0.648 |  |
|  | 10 | control | 123.2 | 0.032 | 0.779 |  |
|  | 11 | grafted | 206.3 | 0.025 | 0.575 | 0.8673 |
|  | 12 | grafted | 155.8 | 0.028 | 0.833 | 0.8046 |
| 4 | 13 | control | 134.3 | 0.029 | 0.609 |  |
|  | 14 | control | 139.4 | 0.024 | 0.647 |  |
|  | 15 | grafted | 172.3 | 0.024 | 0.630 | 1.1449 |
|  | 16 | grafted | 139.5 | 0.022 | 0.722 | 0.5248 |
| 5 | 17 | grafted | 164.5 | 0.026 | 0.573 | 0.7613 |
|  | 18 | grafted | 172.7 | 0.028 | 0.518 | 0.6487 |
|  | 19 | control | 115.9 | 0.030 | 0.514 |  |
|  | 20 | control | 157.7 | 0.024 | 0.580 |  |
| 6 | 21 | control | 160.62 | 0.023 | 0.648 |  |
|  | 22 | control | 167.68 | 0.025 | 0.596 |  |
|  | 23 | grafted | 175.29 | 0.027 | 0.585 | 0.6393 |
|  | 24 | grafted | 147.91 | 0.028 | 0.565 | 0.7530 |
| 7 | 25 | control | 165.3 | 0.028 | 0.598 |  |
|  | 26 | control | 168.2 | 0.026 | 0.544 |  |
|  | 27 | grafted | 142.7 | 0.028 | 0.429 | 0.9134 |
|  | 28 | grafted | 203.8 | 0.023 | 0.623 | 0.7621 |
| 8 | 29 | control | 195.6 | 0.022 | 0.578 |  |
|  | 30 | control | 136.2 | 0.025 | 0.668 |  |
|  | 31 | grafted | 190.8 | 0.026 | 0.558 |  |
|  | 32 | grafted | 207.4 | 0.025 | 0.736 | 0.7221 |
| 9 | 33 | control | 134.1 | 0.031 | 0.600 |  |
|  | 34 | control | 150.1 | 0.023 | 0.578 |  |
|  | 35 | grafted | 209.2 | 0.025 | 0.501 | 1.1090 |
|  | 36 | grafted | 143.9 | 0.027 | 0.624 | 0.9842 |
| 10 | 37 | control | 165.4 | 0.026 | 0.486 |  |
|  | 38 | control | 173.6 | 0.022 | 0.624 |  |
|  | 39 | grafted | 208.9 | 0.020 | 0.576 | 1.5140 |
|  | 40 | grafted | 219.2 | 0.021 | 0.637 | 1.2424 |

Data set 2 : Experimental grafting design

| Donor oyster | Recipient oyster n° |
| --- | --- |
| E | 3 |
| E | 4 |
| E | 32 |
| A | 7 |
| A | 8 |
| A | 11 |
| A | 12 |
| A | 17 |
| A | 18 |
| A | 35 |
| A | 36 |
| B | 15 |
| B | 16 |
| B | 23 |
| B | 24 |
| B | 39 |
| B | 40 |
| C | 27 |
| C | 28 |
